# Sublingual Dermoid Cyst in Young Child

**DOI:** 10.3390/children10020254

**Published:** 2023-01-30

**Authors:** Masakazu Hamada, Rena Okawa, Katsuhiko Masuda, Fumikazu Tojo, Yuto Suehiro, Yuko Ogaya, Hiroko Kinosada, Narikazu Uzawa, Kazuhiko Nakano

**Affiliations:** 1 Department of Oral and Maxillofacial Surgery II, Osaka University Graduate School of Dentistry, Osaka 565-0871, Japan; 2 Department of Pediatric Dentistry, Osaka University Graduate School of Dentistry, Osaka 565-0871, Japan

**Keywords:** dermoid cyst, infant, floor of the mouth

## Abstract

A dermoid cyst is a benign congenital lesion of ectodermal origin that can arise in any region of the body, though occurrence is rare. A young girl aged 2 years 4 months was referred to our hospital because of a painless mass in the floor of the mouth. Intraoral examination findings revealed a painless movable elastic soft mass on the floor of the mouth measuring approximately 15 mm in diameter. Magnetic resonance imaging indicated a cystic lesion, with low signal intensity shown in T1-weighted and extremely high signal intensity in T2-weighted images. These clinical findings indicated the presence of a dermoid cyst and removal was planned. Under general anesthesia with nasal intubation, surgical removal was performed through an incision on the floor of the mouth. Blunt dissection exposed the integrity of the cyst capsule, which was weakly attached to adjacent tissue. The excised mass was 19 mm × 14 mm × 11 mm in size. Histological examination findings confirmed a diagnosis of dermoid cyst. The operation was successfully completed without any complications and the postoperative course was good. It is important to properly evaluate cysts in children and provide proper treatment with appropriate timing.

## 1. Introduction

A dermoid cyst is a benign congenital lesion with an ectodermal origin that can arise in any region of the body, though occurrence is rare [1,2,3]. This type of cyst is slow growing and thought to arise from both congenital and acquired sources [4,5]. Occurrence in the oral cavity accounts for only 0.01% of all oral cysts and 1.6% of all dermoid cysts [2,6,7], with the most common site there reported to be the midline point in the floor of the mouth [2,8]. The floor of the mouth is one of the most common sites for this type of cyst, though it can also appear on a lip or cheek, or the gingiva/alveolar ridge [9,10]. One of those reports [10] also noted that approximately half of affected patients visited their clinic for the first time because they noticed a swelling or mass, while 17.9% came because of an increase in swelling or mass, or a swelling or a mass was initially pointed out. In Japan, medical checkups are widely available and provide an opportunity to receive examinations that can reveal such conditions.

Dermoid cysts are often misdiagnosed as an inflammatory pseudocyst (ranula) or lymphatic malformation [11]. Clinical differential diagnosis includes lymphoepithelial cyst, thyroglossal cyst, ranula, cystic hygroma, lymphangioma, and soft tissue abscess [3]. Standard treatment for a dermoid cyst in the floor of the mouth is surgical removal [2,12].

Most dermoid cysts in the floor of the oral cavity are diagnosed between the ages of 10 and 30 years [3,13]. Occurrence is rarely noted in children and especially rare in infants. Here, we present details of a sublingual dermoid cyst in a young child. Informed consent was obtained from the parents of the patient for publication of this case report and accompanying images.

## 2. Case Presentation

**Figure 1:**
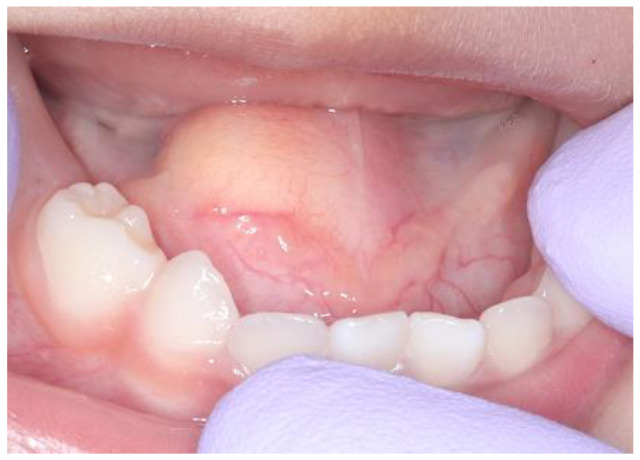
Intraoral photograph obtained at initial visit.

**Figure 2:**
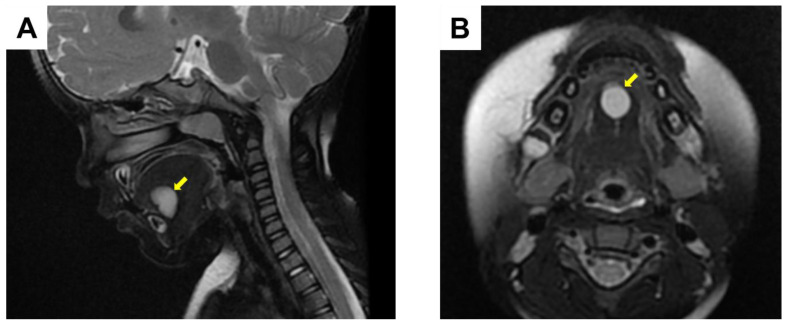
MRI results at time of initial visit. Arrow indicates cystic lesion. (**A**) Axial T2-weighted image. (**B**) Coronal T2-weighted image.

A Japanese girl aged 2 years 4 months was referred to the Department of Pediatric Dentistry and Department of Oral and Maxillofacial Surgery II of Osaka University Dental Hospital for a painless mass in the floor of the mouth. Her mother had been aware of the lesion from the age of approximately one year. A dental checkup was also performed at her nursery school, and they advised that the child should be examined by a specialist. There were no abnormal extraoral findings, while an intraoral examination revealed a painless movable elastic soft mass measuring approximately 15 mm in diameter on the floor of the mouth (Figure 1). No tongue movement disorder or oral intake disorder was observed. Following administration of TRICLORYL Syrup^®^ (triclofos sodium) as a sedative agent, an internal magnetic resonance imaging (MRI) examination was performed, which revealed a cystic lesion with low signal intensity in T1-weighted images and extremely high intensity in T2-weighted images (Figure 2A,B). These clinical findings indicated the presence of a dermoid cyst and removal was planned. 

**Figure 3:**
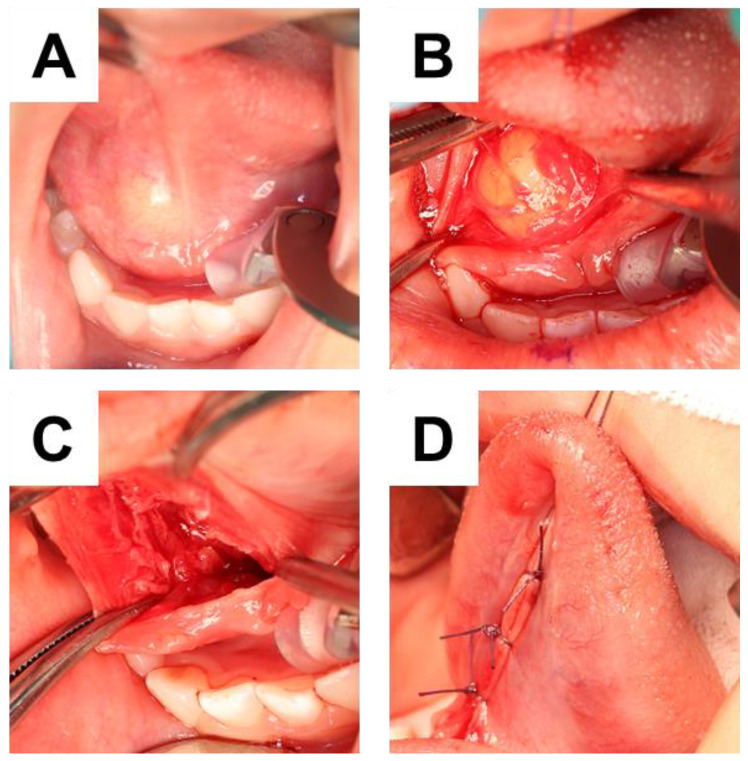
Intraoperative appearance. (**A**) Before operation. (**B**) Lesion appearance after incision. (**C**) After removal. (**D**) After suturing.

**Figure 4:**
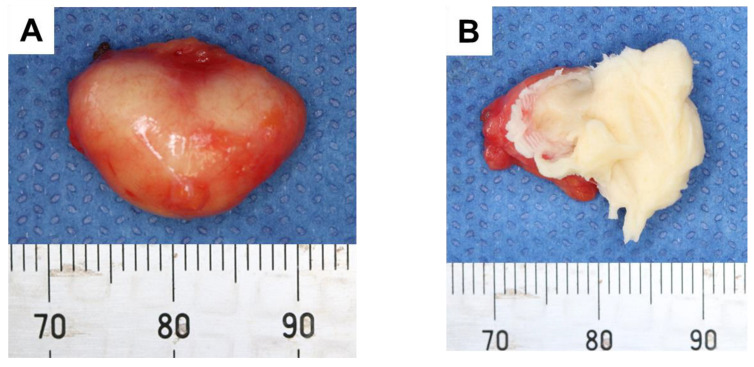
Macroscopic characteristics of cyst. (**A**) After removal. (**B**) Contents of cyst after splitting.

**Figure 5:**
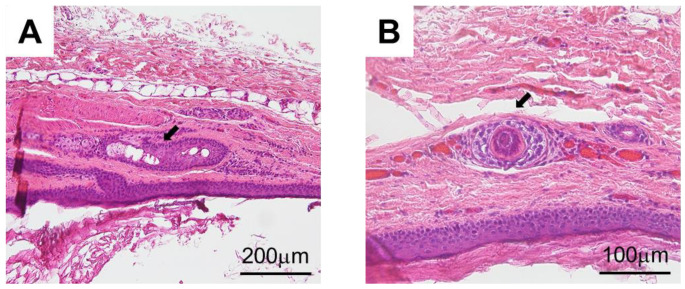
Histopathological examination images (hematoxylin-eosin staining). (**A**) Sebaceous gland (arrow). (**B**) Hair follicle (arrow).

Under general anesthesia with nasal intubation, surgical excision was implemented with an incision on the floor of the mouth (Figure 3A). A blunt dissection exposed the integrity of the cyst capsule, which was weakly attached to adjacent tissue (Figure 3B–D). The excised cyst was elastic soft, and the split surface showed white xerophilic contents (Figure 4). The size was 19 × 14 × 11 mm (Figure 4A), and the cyst contents consisted of lamellated layers of keratin showing desquamation by the epithelial lining (Figure 4B). Histological examination results confirmed a diagnosis of dermoid cyst. The wall of the mass was found to be composed of keratinized stratified squamous epithelium (Figure 5), and the inside was filled with keratinized tissue. Surrounding the cyst were sebaceous glands (Figure 5A) and hair follicles (Figure 5B). The operation was successfully performed without any complications and the postoperative course was good. 

## 3. Discussion

**Figure 6:**
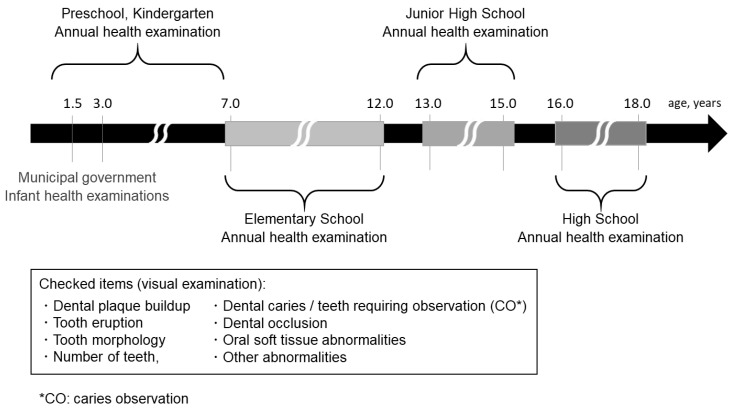
Health examination system for children in Japan.

Regarding the reason for going to a clinic, it is often difficult for the patient themself to notice a mass indicating a dermoid cyst [10]. That study found that approximately 65% of affected patients had symptoms for 5 years or less, though about 20% demonstrated a duration of 10 or more years. Based on the stated motivation for the initial visit and symptom duration, that study concluded that affected individuals are less likely to notice a dermoid cyst, while even if they do, they tend to not request an examination due to its slow growth. In Japan, health examinations are obligatory for children aged 1 year 6 months and 3 years and conducted by municipal governments based on the Maternal and Child Health Act. Furthermore, the Child Welfare Act requires preschools to conduct health examinations every year, while the School Health and Safety act states that kindergartens, elementary schools, junior high schools, and high schools conduct annual health examinations as well (Figure 6). In this context, dental checkups are also performed, during which visual examinations for dental plaque buildup, tooth eruption, tooth morphology, and number of teeth, as well as dental caries and affected teeth requiring observation (CO: caries observation), dental occlusion, oral soft tissue abnormalities, and other abnormalities are included. Oral diseases in childhood tend to affect hard tissues, such as dental caries, tooth morphology, and dental occlusion. However, oral examinations for infants are often difficult because of lack of patient cooperation, thus a quick examination is desirable, which can sometimes make it difficult to notice soft tissue abnormalities. As for the present patient, a painless mass in the floor of the mouth was noted at a nursery school dental checkup, and the mother was recommended to take her to a dental clinic. Regardless of the circumstances, it is important to carefully check soft as well as hard tissues during a dental examination.

Dermoid cysts are benign congenital lesions of ectodermal origin that can occur in any region of the body [1,2,3]. They are slow growing and thought to arise from both congenital and acquired sources [4,5]. The most widely accepted theory proposes a congenital origin, caused by ectoderm movement during fetal life, while another proposes that a dermoid cyst is acquired through trauma or other similar cause [4]. The orbit is the most commonly affected site in the head and neck region [14]. As for occurrence in the oral cavity, the percentage of dermoid cysts has been reported to be only 0.01% of total oral cysts and 1.6% of total dermoid cysts [2,6,7]. In the present patient, the lesion was located in the floor of the mouth. In addition, it was thought to be congenital, because her mother had noticed it since the age of 1 year.

Dermoid cysts are often misdiagnosed as an inflammatory pseudocyst (ranula) or lymphatic malformation [11]. When the patient is a young child, it is difficult to examine the oral cavity because of cooperation management. Clinical differential diagnosis of a dermoid cyst includes lymphoepithelial cyst, thyroglossal cyst, ranula, cystic hygroma, lymphangioma, and soft tissue abscess, with MRI, computed tomography, and ultrasonography examinations helpful for establishing diagnosis [3,15]. As for MRI, T1-weighted images show variations in intensity, while T2-weighted images have a hyperintense appearance [4]. In the present case, the possibility of ranula was initially noted and the patient was referred to our hospital. We were able to perform MRI by administering triclofos sodium before the examination. Consistent with that previous study, the cystic lesion showed low signal intensity in T1-weighted images, while it showed extremely high intensity in T2-weighted images. Based on the location of the lesion in the midline of the floor of the mouth and these MRI findings, it was differentiated from a ranula, and the diagnosis was dermoid cyst.

Standard treatment for a dermoid cyst on the floor of the mouth is surgical removal [2,6]. Based on the location of the lesion and mylohyoid muscle, a determination must be made whether to use an intraoral or extraoral approach [2,12,16]. Furthermore, depending on the size of the cyst, increased salivation, speech disturbance, dyspnea, and difficulties with eating, swallowing, and breathing may be present [17]. Early treatment is required to remove a dermoid cyst, especially when the airway is compromised or swallowing difficulties are noted [8]. Size increases with age until the 30s, and the maximum diameter of the lesion noted in examinations will also become larger over time [18]. In the present case, there were no difficulties with eating or speech disturbance. However, because the size of the cyst was likely to gradually increase in the future, even though there was no eating or speech disturbance, symptoms were also anticipated to appear as the lesion grew. The surgery was scheduled soon after the initial visit, because her mother was due to give birth four months later. An intraoral approach was selected based on the location of the lesion with reference to the mylohyoid muscle.

Recurrence of a dermoid cyst or malignant transformation to a squamous cell carcinoma is extremely rare, and a postoperative infection is also unlikely to occur [16,19]. In the present case, the cyst was easily detached with no adhesions to the surrounding area and removed as a single undamaged mass. No postoperative infection was observed. Although recurrence is unlikely, careful follow-up examinations are considered necessary.

## 4. Conclusions

Increased salivation, speech disturbance, dyspnea, and difficulties with eating, swallowing, and breathing may be present in a patient with a dermoid cyst, which are dependent on its size. Early treatment for removal is generally required, especially when the airway is compromised or swallowing difficulties are noted. When a cyst is noted in a child patient, proper evaluation and treatment at an appropriate time are essential.

## Data Availability

Not applicable.

## References

[1] Lin H.W., Silver A.L., Cunnane M.E., Sadow P.M., Kieff D.A. (2011). Lateral dermoid cyst of the floor of mouth: Unusual radiologic and pathologic findings. Auris Nasus Larynx.

[2] Vélez-Cruz M.E., Gómez-Clavel J.F., Licéaga-Escalera C.J., Montoya Pérez L.A., Trujillo Fandiño J.J., Trejo Iriarte C.G., Ramírez-Cano M.F., García-Muñoz A. (2020). Sublingual dermoid cyst in an infant: A case report and review of the literature. Clin. Case Rep..

[3] Oluleke O.O., Akau K.S., Godwin A.I., Kene A.I., Sunday A.O. (2020). Sublingual Dermoid Cyst: Review of 14 Cases. Ann. Maxillofac. Surg..

[4] Sauer A., Abrol A., Cabrera C.I., Shah J. (2021). A Pediatric Lateral Submental Mass: A Rare Presentation of Dermoid Cyst. Ear Nose Throat J..

[5] Meyer I. (1955). Dermoid cysts (dermoids) of the floor of the mouth. Oral Surg. Oral Med. Oral Pathol..

[6] Lima S.M., Chrcanovic B.R., de Paula A.M., Freire-Maia B., Souza L.N. (2003). Dermoid cyst of the floor of the mouth. Sci. World J..

[7] Rajayogeswaran V., Eveson J.W. (1989). Epidermoid cyst of the buccal mucosa. Oral Surg. Oral Med. Oral Pathol..

[8] Bhalla S., Acharya V., Ally M., Taghi A. (2019). Acute presentation of an intraoral dermoid cyst causing airway compromise in a young child. BMJ Case Rep..

[9] Santos H.B., Rolim L.S., Barros C.C., Cavalcante I.L., Freitas R.D., Souza L.B. (2020). Dermoid and epidermoid cysts of the oral cavity: A 48-year retrospective study with focus on clinical and morphological features and review of main topics. Med. Oral Patol. Oral Cir. Bucal.

[10] Kobayashi Y., Kino K., Manita H., Satou R., Kikuchi K., Yoshimasu H., Amagasa T. (1998). Clinical Observation of Dermoid Cyst and Epidermoid Cyst in the Oral and Maxillofacial Region. J. Jpn. Stomatol. Soc..

[11] Misch E., Kashiwazaki R., Lovell M.A., Herrmann B.W. (2020). Pediatric sublingual dermoid and epidermoid cysts: A 20-year institutional review. Int. J. Pediatr. Otorhinolaryngol..

[12] Ohta N., Watanabe T., Ito T., Kubota T., Suzuki Y., Ishida A., Kakehata S., Aoyagi M. (2012). A case of sublingual dermoid cyst: Extending the limits of the oral approach. Case Rep. Otolaryngol..

[13] Di Francesco A., Chiapasco M., Biglioli F., Ancona D. (1995). Intraoral approach to large dermoid cysts of the floor of the mouth: A technical note. Int. J. Oral Maxillofac. Surg..

[14] Pryor S.G., Lewis J.E., Weaver A.L., Orvidas L.J. (2005). Pediatric dermoid cysts of the head and neck. Otolaryngol. Head Neck Surg..

[15] Akinosi J.O. (1974). Multiple sublingual dermoid cysts. Br. J. Oral Surg..

[16] Liu N.N., Zhang X.Y., Tang Y.Y., Wang Z.M. (2020). Two sequential surgeries in infant with multiple floor of the mouth dermoid cysts: A case report. World J. Clin. Cases.

[17] Şimşek-Kaya G., Özbudak İ.H., Kader D. (2018). Coexisting sublingual dermoid cyst and heterotopic gastrointestinal cyst: Case report. J. Clin. Exp. Dent..

[18] Muratsu D., Kawano S., Minamizato T., Tanaka A., Hashiguchi Y., Kiyoshima T., Shiratsuchi Y., Ikebe T., Nakamura S. (2014). A large dermoid cyst of sublingual type in the oral floor of a child. Pediatr. Oral Maxillofac. Surg..

[19] Dillon J.R., Avillo A.J., Nelson B.L. (2015). Dermoid Cyst of the Floor of the Mouth. Head Neck Pathol..

